# A comparison of the Doppler ultrasound interpretation by student and registered podiatrists

**DOI:** 10.1186/1757-1146-6-25

**Published:** 2013-07-13

**Authors:** Maria Young, Ivan Birch, Chloe Alexa Potter, Robert Saunders, Simon Otter, Shahin Hussain, Jane Pellett, Nadine Reynolds, Sarah Jenkin, Wendy Wright

**Affiliations:** 1 School of Health Professions, University of Brighton, Robert Dodd building 49 Darley Road, Eastbourne BN20 7UR, UK; 2 Sheffield Teaching Hospitals NHS Foundation Trust, Jordanthorpe Health Centre, 1 Dyche Close, Sheffield S8 8DJ, UK

## Abstract

**Background:**

Hand held Doppler ultrasound machines are routinely used by podiatrists to assess the arterial perfusion of the lower limb. They are practical, painless and effective as a screening tool, and the available general evidence would suggest that interpretation by practitioners is reliable. This study compared the abilities of student and Health and Care Professions Council (HCPC) registered podiatrists to identify correctly Doppler ultrasound outputs.

**Method:**

A prospective single blind comparative study design was utilised. Fifteen Doppler recordings of the blood flow in the posterior tibial artery, five each of monophasic, biphasic and triphasic blood flow, were used to compare the interpretation abilities of 30 undergraduate podiatry students and 30 HCPC registered podiatrists. Chi-squared analysis of the results was undertaken.

**Results:**

Chi-squared analysis found that there was no statistically significant difference between the overall abilities of student podiatrists and HCPC registered podiatrists to identify correctly Doppler ultrasound recordings (p = 0.285). No significant difference was found in their ability to identify Doppler ultrasound recordings of monophasic, biphasic or triphasic blood flow (p > 0.050).

**Conclusion:**

The results of this relatively small study suggest that both student and HCPC registered podiatrists are in general able to identify the nature of blood flow based on the output of handheld Doppler ultrasound units. However, the results raise an issue regarding professional development of practitioners who might have been expected to have enhanced their skills of Doppler ultrasound sound identification since professional registration.

## Background

Originally developed by Christian Doppler in the 19th Century, Doppler waveform has now become a portable and easily usable method of establishing pulse wave-form and systolic blood pressures [1]. Assessment of the arterial perfusion of the lower limb using hand held Doppler ultrasound machines is practical, painless and effective [2,3]. As a component of a multimodal approach to vascular assessment it has been used routinely by podiatrists since its validation as a clinical screening tool in the 1980s [4] for both the detection of peripheral arterial disease (PAD), and to categorise the risk of a patient developing complications such as ulceration, gangrene and amputation [5]. Doppler ultrasound investigation is particularly effective in the calculation of ankle brachial pressure indices and the assessment of blood pressures in the toe, and has become a valuable tool in PAD detection for patients with diabetes [6] as the non-invasive nature of Doppler ultrasound investigation poses minimal risk to the patient whilst providing accurate diagnostic capability [7].

Two main pulse sites are commonly used by podiatrists during vascular assessment, the posterior tibial and dorsalis pedis arteries [8]. The Doppler probe is placed over the pulse sites, with acoustic gel applied, and angled so that the emitted ultrasound waves are projected obliquely into the oncoming blood flow [9]. The ultrasound waves are reflected by the blood cells back to the probe and Doppler ultrasound machine, which produces an audible output. Three patterns of blood flow, producing characteristic audio outputs from the Doppler ultrasound machine, are commonly associated with vascular assessment [10]:

triphasic which occurs in arteries and is the result of the combination of ventricular systole, elasticity of the blood vessels and the backflow caused by the closing of the semilunar valves;

biphasic which occurs in more distal blood vessels as the result of ventricular systole and the elasticity of the blood vessels;

monophasic which occurs when the flow of blood is no longer pulsatile.

All three types of blood flow occur in healthy subjects. However, the transition over time from one type to another in a particular blood vessel and location is a significant diagnostic indicator of changes in the effectiveness of the circulatory system.

If standard protocols and techniques are applied, the Doppler is relatively error free when used to help identify the type of blood flow [11]. The classification of the blood flow based on these three outputs, used in conjunction with other diagnostic techniques, helps the clinician to assess for the presence of peripheral vascular disease, the patient’s risk status, and provides a method of detecting and monitoring deterioration. Doppler ultrasound assessment can therefore have an important impact on a patient’s access to podiatry services in the NHS.

In a systematic review by Mawah et al. [12] it was found that the weakness in the use of Doppler ultrasound assessment in clinical practice was the interpretation of the audible output by the clinician. The review highlighted not only the risk of litigation and economic waste, but more importantly the lack of treatment received by critically ill patients due to misdiagnosis based on misinterpreted Doppler ultrasound outputs. Inaccurate interpretation of Doppler ultrasound outputs by podiatrists could therefore contribute to the sub-optimal management of PAD or conversely subject patients to unnecessary investigation [13].

A clinical assessment tool is only of value if the interpretation of the results is correct and repeatability has been clinically established [14]. Review of the literature shows that poor training, inexperience and time constraints may contribute to erroneous interpretation of Doppler ultrasound outputs [10,13,15], and that inter and intra-observer reliability is variable and experience and technique dependent [4]. Studies have found that qualified staff trained in vascular assessment are more accurate than junior staff of their relative professions in Doppler sound interpretation [13,15,16], although none of these studies looked specifically at Doppler sound interpretation by podiatrists. However, in view of the findings of these studies, experienced podiatry practitioners might be expected to be more adept at Doppler ultrasound interpretation than less professionally experienced students. Tweedie [10] suggested that a diagnostic tool used as regularly as podiatrists use the hand held Doppler ultrasound should be subject to profession specific research.

This study compared the Doppler ultrasound sound interpretation abilities of HCPC registered podiatrists with that of 2^nd^ and 3^rd^ year student podiatrists on a BSc (Hons) Podiatry programme.

## Method

Ethical approval was obtained from the Research Ethics Committee of the School of Health Professions, University of Brighton. A convenience sample of nine consenting patients were recruited for the recording of the Doppler ultrasound sounds, three patients displaying a monophasic, three a biphasic and three a triphasic blood flow. Patients were excluded if they were under the age of 18, unable to give informed consent, did not have Doppler detectable pulses or had a known allergy to the coupling gel.

Each patient was placed in a quiet room and an 8 MHz Doppler probe held over the posterior tibial artery of the left foot and the audible output from Doppler ultrasound machine recorded for 20 seconds using a digital Dictaphone. All recordings were collected by the same researcher, using the same Doppler ultrasound machine, and recorded using the same digital Dictaphone. In order to standardise recording volume the distance of the Dictaphone from the machine’s speaker was the same for all recordings. A graph of the waveform, produced by the Doppler ultrasound machine, was also collected for each patient. Each of the nine graphs was scrutinised and interpreted by the research group (CAP, RS, SH, JP, NR, SJ, WW), using the manufacturer’s guidance, to ensure accurate classification of the audible output. The interpretation was then independently verified by an experienced podiatrist (MY). Two duplicate recordings were also made of each of one of the monophasic, biphasic and triphasic recordings, giving a total of fifteen 20 second recordings, all of which were used in the study. This duplication of recordings was undertaken to allow the potential for future investigation into the repeatability of interpretation.

A prospective single blind comparative study design was utilised. Thirty students in the 2^nd^ and 3^rd^ year of the BSc Honours programme at the University of Brighton, were recruited using posters. The combination of 2^nd^ and 3^rd^ year students was used in light of preliminary investigations undertaken at the University of Brighton, which had shown that there was no significant difference (p = 0.933) between the Doppler ultrasound interpretation abilities of members of these two year groups. Thirty HCPC registered podiatrists were recruited following a short presentation during a local continuing professional development (CPD) meeting of members of the Society of Chiropodists and Podiatrists UK. All participants were provided with full information and written consent obtained. Participants were excluded if they reported having any hearing impairments, operator hearing impairment being cited in the literature as a reason for the inaccurate interpretation of Doppler sounds [2,8].

In turn, participants were placed in a small study room with the door closed. The participant put on the headphones provided and was played a prepared CD through Windows Media Player 10 at a volume of 75/100. The CD had a short introduction of instructions, followed by the fifteen Doppler ultrasound recordings, ordered randomly using a random number generator. A ten second interval between recordings allowed the participant time to make a judgement about the sound and record on a data sheet whether the Doppler ultrasound recording was of a monophasic, biphasic or triphasic blood flow.

The data sheets were collated to give a matrix of student and HCPC registered podiatrists’ results. Descriptive statistics were produced for all of the data, and a chi square analysis undertaken to test for statistically significant differences between the two groups.

## Results

Only two participants, both students, interpreted all fifteen Doppler ultrasound sounds correctly. Table 1 shows the descriptive statistics derived from the data from the two groups. This shows students and HCPC registered podiatrists to have produced comparable results.

**Table 1:** Descriptive statistics derived from the scores of individual students and HCPC registered podiatrists (maximum possible score 15)

	**Students**	**HCPC Registered Podiatrists**
Minimum correct	6	2
Maximum correct	15	14
Mode	14	14
Median	13	13
Mean	12.6	12.2

The recordings of monophasic blood flow were identified most reliably (on 90% of occasions by both student and HCPC registered podiatrists,) and the recordings of triphasic blood flow least reliably (74% and 69% respectively). Table 2 shows the identification data produced by students and HCPC registered podiatrists for each of the three types of Doppler sound (mono, bi and triphasic) analysed using chi squared analysis.

**Table 2:** Group outcomes of students and HCPC registered podiatrists for each of the three types of Doppler sound based on chi squared analysis

	**Students (correctly identified)**	**HCPC Registered Podiatrists (correctly identified)**	**chi square test outcome**		
			***χ***^**2**^	**p value**	**outcome**
Monophasic	135/150 (90%)	135/150 (90%)	0.00	1.000	no significant difference
Biphasic	134/150 (89%)	129/150 (86%)	0.77	0.379	no significant difference
Triphasic	111/150 (74%)	104/150 (69%)	0.80	0.369	no significant difference
Combined	380/450 (84%)	368/450 (82%)	1.14	0.285	no significant difference

The chi squared analysis showed there to be no significant difference (p = 0.285) in the overall ability of student podiatrists and HCPC registered podiatrists to identify correctly Doppler ultrasound sound recordings of the blood flow in the posterior tibial artery. No significant difference was also shown in the ability to identify correctly monophasic, biphasic or triphasic sound recordings.

## Discussion

The results show that there is no statistically significant difference between the ability of student podiatrists and HCPC registered podiatrists to identify correctly Doppler ultrasound sound recordings of the blood flow in the posterior tibial artery. Furthermore, both groups showed mean correct identification rates of better than 12 out of 15, suggesting reasonable identification abilities. This is somewhat contrary to the findings of similar studies [13,15,16]. These all showed qualified professionals produced better results than less experienced healthcare workers during different vascular assessment skills.

In view of the potential, or even expectation, for the enhancement of diagnostic skills, such as the use of Doppler ultrasound, through a combination of mandatory CPD and clinical experience, the results of this study raise an interesting question. Why were the practitioners not better than the students? The disagreement with the findings with those of previous studies [13,15,16] with regard to the lack of difference in performance between qualified professionals and less experienced healthcare workers, could be the result of a number of factors. The registered participants were drawn from a professional body local branch meeting and all were HCPC registered. However, not all had qualified in podiatry through the three/four year diploma or degree route, and there was therefore no guarantee that each practitioner had received training in the use of a Doppler machine. The sample of registered participants included both NHS based and private practice based practitioners. There was therefore a variation in access to training and equipment, and in the frequency with which Doppler ultrasound was used as a standard assessment tool. A number of private practice based practitioners either did not possess, or rarely used a Doppler ultrasound hand held machine for screening purposes. While the practitioner sample showed variation in the frequency of use of Doppler ultrasound, the student sample did not, students using Doppler ultrasound as an assessment tool regularly and frequently. The use of student podiatrists rather than other less experienced healthcare workers could also therefore have contributed to the discord with previous findings. In view of the findings of this study, further research should be undertaken to investigate the differences in Doppler ultrasound interpretation by practitioners at different stages of their career, taking into account variations in training and CPD.

Patterns of correct identification for the three types of blood flow were seen to be the same for both groups, with monophasic sounds being most reliably identified, and triphasic the least reliably identified by both groups. These findings are contrary to those of previous studies by Vowden et al. [11] and Lithgoe and Barlow [7], who found biphasic pulses to be the most inconsistently interpreted sounds.

Some practitioners expressed concern about their own competence in the use of Doppler ultrasound and the interpretation of the sounds produced, citing a lack of knowledge, training and/or experience as reasons. All practitioners were recruited through attendance at the local Society branch meeting, and were therefore engaged in continuing professional development. Nevertheless, this raises concern over the competence of some practitioners to screen for PAD, and their role as holistic care practitioners [17]. With increasing prevalence of high risk conditions such as peripheral vascular disease and type 2 diabetes, the results of this study identify a particular learning need within the podiatry community. Continuing professional development is mandatory under the Health and Care Professions Council Code of Conduct, and this study provides evidence of a need to offer support and education to practitioners who have rarely used, or have never been trained to use, a hand held Doppler ultrasound machine.

The study design minimised the number of uncontrolled variables. By using the same consulting room, headphones and computer software to play the Doppler audio sounds, every participant was exposed to the same environmental conditions, sound quality and recording order. However, the participants were taken out of their natural clinical setting and the test performed was in isolation of other information usually available to the podiatrist regarding the vascular status of the patient.

## Conclusion

The results of this study show that both student and HCPC registered podiatrists are in general able to identify the nature of blood flow based on the output of a handheld Doppler ultrasound machine. These results raise issues regarding the role of CPD and clinical experience in the enhancement of diagnostic skills, and highlight the need for support and education for practitioners who may rarely use or have never been trained to use a hand held Doppler ultrasound machine. While the sample size employed in this study provides a sound basis for the findings and conclusions, a larger study, taking account variations in professional experience, training and CPD is warranted to inform the enhancement of professional education at all stages of career development.

## Competing interests

The authors declare that they have no competing interests.

## Authors’ contributions

CAP, RS, SH, JP, NR, SJ and WW administered and carried out the collection of Doppler ultrasound recordings from patients and interpretation data from participants. MY and SO guided the research design, supervised the research and provided interdependent validation of the recordings and results. IB guided the interpretation of the results, undertook the data analysis and produced the final manuscript. All authors read and approved the final manuscript.
